# Rumen-Protected Glucose Stimulates the Insulin-Like Growth Factor System and mTOR/AKT Pathway in the Endometrium of Early Postpartum Dairy Cows

**DOI:** 10.3390/ani10020357

**Published:** 2020-02-23

**Authors:** Yan Wang, Xuefeng Han, Zhiliang Tan, Jinhe Kang, Zheng Wang

**Affiliations:** 1 CAS Key Laboratory for Agro-Ecological Processes in Subtropical Region, National Engineering Laboratory for Pollution Control and Waste Utilization in Livestock and Poultry Production, Hunan Provincial Key Laboratory of Animal Nutrition & Physiology and Metabolism, Institute of Subtropical Agriculture, The Chinese Academy of Sciences, Changsha 410125, China; wangyehaol@163.com (Y.W.); xfhan@isa.ac.cn (X.H.); zltan@isa.ac.cn (Z.T.); 2 College of Bioscience and Biotechnology, Hunan Agricultural University, Changsha 410128, China; 3 College of Agriculture and Biotechnology, Hunan University of Humanities, Science and Technology, Loudi 417000, China

**Keywords:** cow, endometrium, involution, rumen-protected glucose

## Abstract

**Simple Summary:**

A rapid recovery of the uterus is important in order to shorten the interval between pregnancies. An insufficient availability of glucose in early lactation can delay the uterine recovery. However, little information is available on dietary rumen-protected glucose addition on the uterine involution and insulin-like growth factor system in the endometrium of dairy cows during the early post-natal period. In this study, dairy cows were fed with 0 or 200 g rumen-protected glucose twice every day from −7 ± 2 to 14 d during the post-natal period. The results indicated that dietary rumen-protected glucose addition promoted the proliferation of endometrial cells in postpartum dairy cows by stimulating the insulin-like growth factor system and rapamycin complex 1 pathway, and might be beneficial for uterine recovery.

**Abstract:**

This study aimed to elucidate the effects of a dietary rumen-protected glucose (RPG) addition on uterine involution through the analysis of an insulin-like growth factor (IGF) system and associated pathways in the post-natal endometrium. Twelve Holstein cows were assigned equally to two groups: a control group (CT) and an RPG group (200 g of RPG per cow per day). The plasma content of insulin-like growth factor 1 (IGF1) was determined by using the ELISA method. Expressions of IGF members, the matrix metalloproteinase, protein kinase B (AKT)/mechanistic target of rapamycin complex1 (mTOR) signaling pathway, and cell proliferation factors (proliferating cell nuclear antigen (PCNA) and Ki67) were detected using real-time polymerase chain reaction, Western blot, immunohistochemistry, and immunofluorescence, respectively. The results showed that the positive cells of PCNA and Ki67 were increased in the endometrium of RPG versus CT cows. The RPG addition significantly increased the plasma IGF1 level 14 d after delivery. The mRNA expressions of the IGF family members (IGF1, IGF2, type 1 IGF receptor (IGF1R) and IGF-binding proteins (IGFBP1, IGFBP2, IGFBP4 and IGFBP5)) were upregulated, and mRNA expressions of matrix metalloproteinase MMP3 and MMP9 were downregulated in cows from the RPG group compared with the CT group. Meanwhile, the protein expressions of IGF1, IGF2, IGF1R, IGFBP1 and IGFBP4 were upregulated in cows from the RPG group compared with the CT group. Immunohistochemical analysis identified a positive response for IGF1R and IGF2R in the endometrium of RPG versus CT cows. Furthermore, the RPG supplementation increased the protein expressions of phosphorylated (p)-AKT to total AKT and p-mTOR to total mTOR ratio in the endometrium. The current results indicated that the RPG supplementation promoted the proliferation of endometrial cells by stimulating the IGFs and mTOR/AKT pathway in the early post-natal endometrium of dairy cows.

## 1. Introduction

Glucose plays an important role in lactation and reproduction [1]. Generally, glucose is rapidly fermented to volatile fatty acids in the rumen, and small amounts of glucose are absorbed in the digestive tract. As a result, glucose is resynthesized by gluconeogenesis in the liver of post-natal cows. However, glucose requirements during early lactation are usually not satisfied by some diets for high-producing dairy cows. The production of each kilogram of milk requires 72 g of glucose [1]. Only 85% of glucose demands for milk production are met by available volatile fatty acid (VFA) during early lactation, resulting in a shortfall of 500 g of glucose per day [2]. Glucose is also required by uterine involution in the postpartum period and provides carbon to synthesize cellular components (such as nucleotides, lipids and so on) in the uterus. The uterine recovery was delayed by insufficient glucose in the early lactation of cows [3], it impairs normal and rapid uterine involution, and thereby decreases reproductive efficiency [4]. In addition, concentration of blood glucose during the early post-natal period can predict subsequent postpartum fertility.

It has been accepted that the rapid recovery of the uterus in the post-natal period is important for achieving short intervals of pregnancy. Uterus involution is mediated by growth factors and matrix-degrading enzymes. An insulin-like growth factor (IGF) system includes IGF and IGF binding proteins (IGFBPs) and is known to assist uterine involution [5,6], principally acting through IGF1R and IGF2R. Moreover, the effects of IGFs in the post-natal uterus are highly regulated by the IGFBPs [7]. The reduction of the plasma IGF1 level in the early post-natal period affects the expressions of tissue repairing-related genes in the endometrium of dairy cows [4]. Furthermore, the recovery of the uterus in the post-natal period is usually accompanied by extensive extracellular matrix remodeling, which is modulated by matrix metalloproteinases (MMPs) [8]. Tissue remodeling is closely related to the proliferation of cells, which is mediated by the AKT/mTOR pathway [9].

In order to enhance direct absorption of glucose from the gastrointestinal tract, it is feasible to selectively supplement carbohydrate resources in diets that can bypass the rumen without easily being fermented. For instance, some cereal grains like sorghum and some corn hybrids contain starch which partially escapes the rumen and can be digested in the duodenum [10], thereby increasing the absorption of glucose. More directly, rumen-protected glucose (RPG) coated with fat has been developed to enhance the absorption of glucose from the gastrointestinal tract of ruminants. Feeding RPG is becoming an alternative strategy to increase glucose and the energy supply for dairy cows. Our previous studies also indicated that RPG plays a key role in improving post-natal lactation and inflammation [11]. However, little information is available on RPG supplementation on the uterine involution and IGF system in the endometrium during the early post-natal period. Thus, the aims of the current study were to investigate the expression profiles of members of the IGF system, MMPs and mTOR/AKT pathway in the endometrium of dairy cows supplemented with RPG from −7 ± 2 to 14 d during the post-natal period.

## 2. Methods and Materials

The experiment was conducted at the Experimental Dairy Farm of Hunan Institute of Animal and Veterinary Science, Changsha, China. The protocol was followed according to the Animal Welfare Committee (Permit No. ISA000257), Institute of Subtropical Agriculture, Changsha, China. 

### 2.1. Animals and Experimental Procedures

Twelve healthy Holstein cows (4–5 years old; 515 ± 42 kg body weight; 16.1 ± 3.7 kg milk production per day) with the second or additional amounts of lactation were enrolled in the study. Cows were randomly assigned to two treatments: (1) control diet (CT) without rumen-protected glucose (RPG) supplementation (*n* = 6); (2) CT plus RPG (Yahe Nutrition Technology Co., Ltd., Beijing, China) (*n* = 6). The RPG addition is produced by a patented technique, which includes 45% glucose coated with fat (45%). The RPG addition is granular and the diameters of the particles were 0.6–0.85 mm. Each cow was fed with 200 g of RPG per day. The same amounts of coating fat were also supplemented in the CT group with 90 g per cow daily to exclude the effect of the fat coated in the RPG.

All cows were fed with the same far-off diet from −50 to −22 d before the expected delivery, close-up diet from −21 d to expected delivery, and lactation diet from delivery through 20 d. The addition of RPG and coating fat on the Total Mixed Rations (TMR) was twice per day from −7 ± 2 to 14 d. The chemical composition and ingredients of the diets are shown in Table 1. Dairy cows were fed twice daily at 7:30 and 14:30 h ad libitum. Water was freely available throughout the trial period.

### 2.2. Sample Collection 

Blood samples of each cow were collected from the jugular vein using heparinized tubes (18 U/mL) 7 d and 14 d after delivery, and immediately stored on ice. Plasma was obtained via centrifugation (3000× *g*, 15 min) and stored at −80 °C. The cows approximately 14 d during the post-natal period were transported to a commercial slaughterhouse and slaughtered by electrical shock method following the usual practices of the China beef industry. The endometrium were collected from cows within 20 min after slaughter, rinsed in RNase-free phosphate buffer, immediately frozen in liquid nitrogen and then stored at −80 °C. The uterine tissues were fixed in 10% buffered formalin solution for histology, immunohistochemistry and immunofluorescence double-labeling analysis.

### 2.3. Immunofluorescence Double-Labeling

The 10% buffered formalin was used to fix the uterine tissues. After being processed routinely through ascending xylene and alcohols, the fixed tissues were embedded in paraffin, and the section thickness was 5 µm. The immunofluorescence was determined according to previous protocols [12], although it was slightly modified. Firstly, the retrieval of the antigen was conducted in 10 mM citrate buffer (pH 6.0, 120 °C, 10 min). Bovine serum albumin (BSA, Service bio, Wuhan, China), 5%, was used to block the nonspecific antigens (30 min, room temperature). Then, the primary antibodies of Ki67 (dilution, 1:200, ab15580, Abcam) and proliferating cell nuclear antigen (PCNA) (dilution, 1:500, GB13010-1, Service bio) were used to incubate the sections (4 °C, overnight). Next, the sections were washed in phosphate buffered saline (PBS) for 15 min (3 × 5 min) and incubated in Alexa Fluor^®^CY3-conjugated goat anti-rabbit IgG (1:300; GB21303, Service bio, Wuhan, China) and Alexa Fluor^®^ 488-conjugated goat anti-mouse IgG (dilution, 1:400; GB25301, Service bio) for Ki67 and PCNA-labeling, respectively (room temperature, 1 h). After being washed with PBS 15 min (3 × 5 min), the sections were stained with 4′,6-diamidino-2-phenylin-dole (DAPI) (Invitrogen, Eugene, OR, USA) at room temperature for 10 min. Finally, the sections were observed under a Nikon eclipse Ci upright fluorescence microscope (Nikon DS-U3 camera, Nikon, Japan). Six microscopic fields were selected randomly (40× magnification), and the numbers of Ki67 or PCNA positive cells were estimated by using the Image-Pro Plus 6.0 software (Version 6.0; Media Cybernetics, Rockville, MD, USA) [13]. More than 300 cells per condition were counted.

### 2.4. Insulin-Like Growth Factor 1 Assay

The concentration of insulin-like growth factor 1 (IGF1) in plasma was determined by a commercial bovine IGF1 ELISA kit (Cusabio Biotech Co., Ltd., Hubei, China). 

### 2.5. Total RNA Extraction and cDNA Synthesis

The Trizol reagent (Invitrogen, Carlsbad, CA, USA) was used to extract the RNA from the endometrium of each cow. The RNA concentration was evaluated with an ND-1000 UV-vis spectrophotometer (NanoDrop Ltd., Wilmington, DE, USA), and the quality of RNA was determined by both 1% agarose gel electrophoresis and the 260/280 absorbanceratio using an ND1000 spectrophotometer (NanoDrop Technologies Inc., Wilmington, DE, USA) [14]. The subsequent experiments only used the RNA samples with a ratio from 1.8–2.1. Next, 5× gDNA Eraser Buffer, 2.0 μL Eraser Buffer, 1.0 μL DNA Eraser and RNase-free DNase I (TaKaRa, Dalian, China) were used to remove the chromosomal DNA in 1 µg of RNA samples (42 °C, 2 min). Thereafter, a reverse transcription was conducted using a PrimeScript^TM^ RT reagent kit (TaKaRa, Japan), and stored at −80 °C for real-time polymerase chain reaction (RT-PCR) analysis.

### 2.6. RT-PCR Analysis

Primers were chosen based on the results from previously published papers [15,16] and listed in Table 2. RT-PCR was conducted by a LightCycler480 system (Roche, Castle Hill, NSW, Australia) using the TB Green^®^ Premix EX Taq^TM^ kit (TaKaRa, Dalian, China). The RT-PCR parameters were as follows: 95 °C for 30 s, then 40 cycles of denaturation (95 °C, 5 s), annealing (60 °C, 20 s), and extension (72 °C, 1 min). The specificity of the PCR reaction was confirmed through a melting curve analysis. The efficiency of PCR amplification for each gene was checked with the dilutions of the samples. The GAPDH was used to normalize the relative expression levels of target genes [16]. The 2^−ΔΔCt^ method was used for data analysis, as previously described [17,18]. 

### 2.7. Western Blot Analysis

After liquid nitrogen grinding, the endometrium samples were homogenized in ice-cold homogenization RIPA buffer, which contained the Protease Inhibitor Cocktail (Roche, Penzberg, Germany) and Phosphatase Inhibitor Cocktail (Roche, Penzberg, Germany). After putting on ice for 30 min, the cell lysates were centrifuged (12,000× *g*, 15 min, 4 °C), and the supernatant was obtained. The protein content of the supernatant was determined with an enhanced BCA protein assay kit (Beyotime Biotechnology, Shanghai, China). Samples were mixed with 5× loading buffer, denatured (100 °C, 5 min), and loaded to 10% sodium dodecyl sulfate-polyacrylamide gel electrophoresis (SDS-PAGE) at 80 V for 30 min, then 120 V for 1.5 h. Then, the isolated proteins were transferred into Polyvinylidene Fluoride membranes (PVDF membrane, Millipore, Danvers, MA) with 0.45 μm apertures at 200 mA for 1.5 h at 4 °C. After transfer, 5% bovine serum albumin (BSA) and 0.1% Tris-Buffered-Saline with Tween (TBST) were used to block the membrane at room temperature for 2 h. The membranes were washed and subsequently incubated with the specific primary antibodies (Table 3) overnight at 4 °C. Then, secondary antibodies (Table 3) were incubated with the membranes (room temperature, 2 h). Finally, the blot was washed with wash buffer (3 × 10 min) and detected by enhanced chemiluminescence (ECL) using Luminata Classico Western HRP Substrate (WBLUC0100, Millipore, Danvers, MA, USA). An imaging system (Bio-Rad, California, USA) was used for the record of the ECL signals and the Quantity One software (Bio-Rad, USA) was utilized for analysis. The protein levels were expressed as a fold change compared with the average value of the CT group.

### 2.8. Histological and Immunohistochemical Assessment

The paraffin-embedded uterine tissues were sectioned at 5 µm. The slides were stained with hematoxylin and eosin (H&E) staining [19] and observed under an Eclipse TE2000U inverted microscope with a twin CCD camera (Nikon, Tokyo, Japan). Subsequently, the expression of IGF1R and IGF2R were detected by using an immunoperoxidase method. Three sections of each tissue for each cow per group were detected according to the published methods [20]. The primary antibodies of IGF1R (Biosynthesis Biotechnology, Beijing, China, 1:100) and IGF2R (Biosynthesis Biotechnology, Beijing, China, 1:400) were used to incubate sections at 4 °C for 24 h. Then, the sections were cleaned in PBS solution (3 × 5 min) and incubated with biotinylated anti-rabbit IgG antibody (Birmingham, AL, USA), and diluted 1:200 in PAV buffer for 50 min at 4 °C. Thereafter, the slides were cleaned in PBS solution (3 × 5 min) and visualized using diaminobenzidine tetrahydrochloride (DAB). Next, the slides were washed and counterstained with Mayer’s hematoxylin. After that, the slides were dried off and covered with a mounting medium (DPX; POCh, Gliwice, Poland). Finally, the slides were detected by three independent observers using an optical microscope.

### 2.9. Statistical Analysis

All the data of this study were expressed as the mean ± standard deviation of the mean (SD). The statistical significance was evaluated by the independent sample *t*-test using the SPSS 21.0 software (IBM; Armonk, NY, USA). *p* < 0.05 indicated significant differences, while 0.05 < *p* < 0.1 indicated a tendency. The Graph Pad Prism software (Version 5.01, La Jolla, CA, USA) was used for graphs.

## 3. Results

### 3.1. Body and Uterine Weight, Plasma IGF1 Concentration 

RPG supplementation did not affect the weights of the body (*p* = 0.28) and uterus (*p* = 0.18), as well as the length (*p* = 0.91) and width (*p* = 0.77) of the uterus in dairy cows 14 d after calving compared with the CT group (Table 4). 

**Figure 1:**
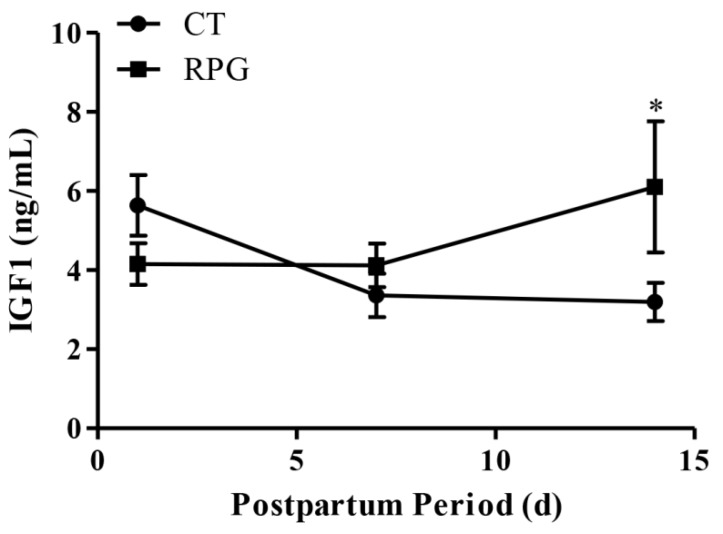
Plasma concentrations of IGF1 in post-natal cows in the RPG and the CT group during the experimental period. Data are presented as the means ± SD. Compared to control diet: * *p* < 0.05.

There were no significant differences (*p* > 0.05) regarding plasma IGF1 concentrations on 1 d and 7 d after delivery, but plasma IGF1 concentrations were higher (*p* < 0.05) in the RPG group on d 14 after delivery, compared to the CT group (Figure 1).

### 3.2. Proliferation Status in the Uterus

**Figure 2:**
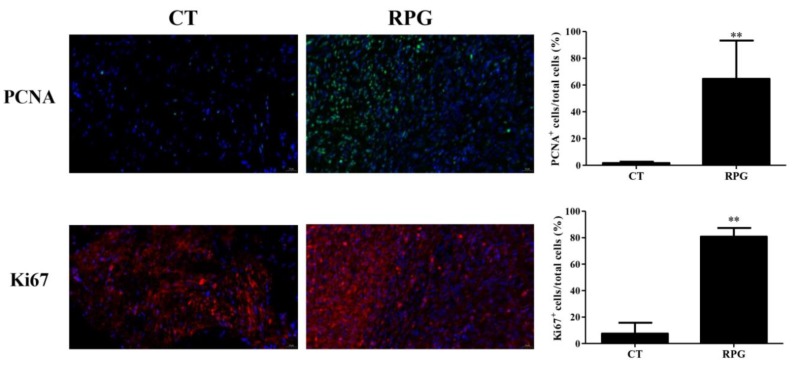
Double-labeling immunofluorescence staining for proliferating cell nuclear antigen (PCNA) (**green**) and Ki67 (**red**) in post-natal dairy cows endometrium. Scale bars = 20 μm. Data are presented as the means ± SD. Compared to control diet: ** *p* < 0.01.

The RPG supplementation induced a significant (*p* < 0.01) increase in PCNA and Ki67-positive cells in the endometrium of cows on 14 d after calving (Figure 2).

### 3.3. Gene Expression Profile 

**Figure 3:**
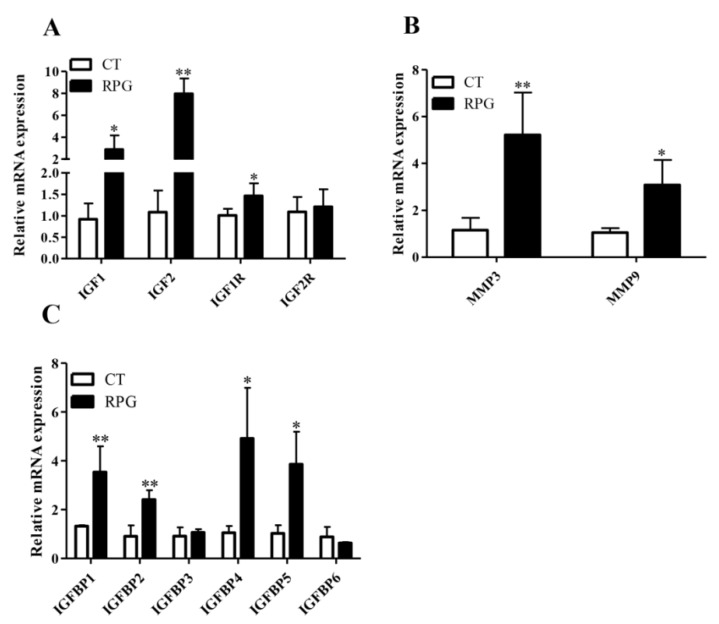
Relative mRNA expression levels for (**A**) IGF1, IGF2, IGF1R, IGF2R, (**B**) MMP3, MMP9 and (**C**) IGFBP1-6 in the endometrium of post-natal dairy cows fed with RPG supplementation compared with the control diet (CT) during the experimental period. Data are presented as the means ± SD. * *p* < 0.05, ** *p* < 0.01.

The endometrial IGFs (IGF1, IGF2 and IGF1R) and IGFBPs (IGFBP1, IGFBP2, IGFBP4 and IGFBP5) mRNA expression levels from the RPG group were significantly higher (*p* < 0.05) than those in the CT group, with trends towards increased IGF2R (*p =* 0.08) (Figure 3A,C). However, the mRNA expression levels of IGFBP3 and IGFBP6 were not changed significantly (*p* > 0.05) in the RPG cows versus CT cows (Figure 3C). The MMP3 and MMP9 gene expressions from RPG cows were significantly higher (*p* < 0.05) than those in CT cows (Figure 3B). 

### 3.4. Functional Protein Expression Pattern

**Figure 4:**
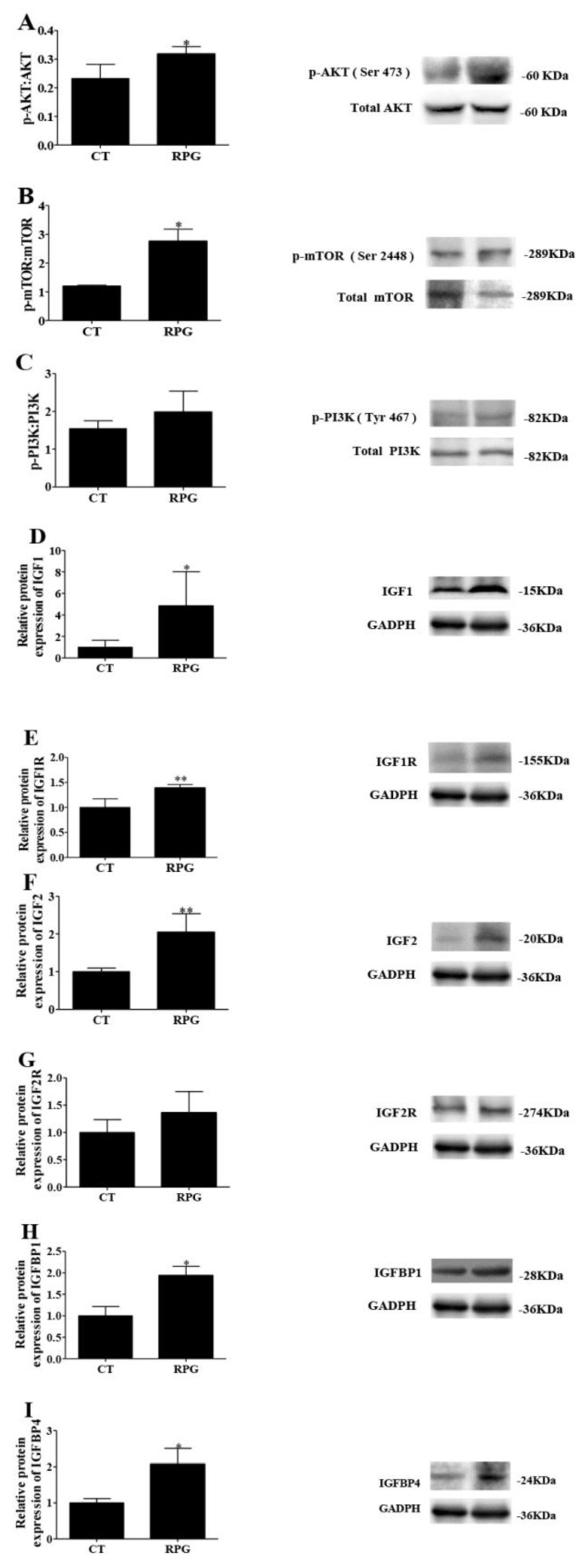
Immunoblotting results for phosphorylated proteins and IGF family members protein expression in uterine tissue from the post-natal cows fed with RPG supplementation and the control diet (CT) during the experimental period. Least squares geometric means of the ratio of phosphorylated (p) protein kinase B (AKT; Ser473): AKT (**A**), p-mechanistic target of rapamycin complex 1 (mTOR, Ser2448): mTOR (**B**), p-phosphatidylinositol 3-kinase (PI3K, Tyr 467): PI3K (**C**), IGF1 (**D**), IGF1R (**E**), IGF2 (**F**), IGF2R (**G**), IGFBP1 (**H**) and IGFBP4 (**I**) proteins are presented. Data are presented as the means ± SD. * *p* < 0.05, ** *p* < 0.01.

The endometrial protein expression of the ratio of p-AKT to total AKT and of p-mTOR to total mTOR increased (*p* < 0.05) in RPG cows versus CT cows (Figure 4A,B). However, the ratio of p-phosphatidylinositol 3-kinase (PI3K) to total PI3K did not change (*p* > 0.05) between the two groups (Figure 4C,G). The protein expression of IGF1, IGF1R, IGF2, IGFBP1, and IGFBP4 increased (*p* < 0.05) in the uterus of RPG cows versus CT cows (Figure 4D–F,H,I). No change (*p* > 0.05) in the protein expression of IGF2R was observed (Figure 4G).

### 3.5. Histopathologic Changes and Immunohistochemical Localization of IGF1R and IGF2R in the Uterus

**Figure 5:**
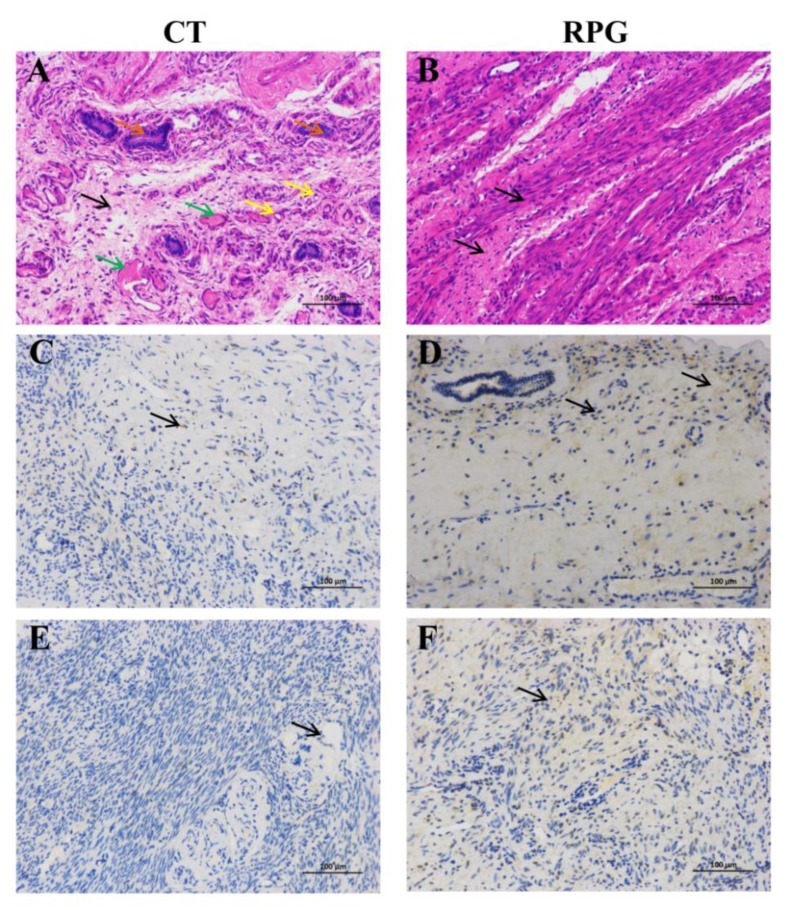
Hematoxylin and eosin (H&E) staining (**A**,**B**) and immunohistochemical analysis of the expression of IGF1R (**C**,**D**) and IGF2R (**E**,**F**) of the endometrium in post-natal dairy cows. In **A**, large amounts of eosin-stained (**orange arrows**) secretions are found in glandular, and blood vessel congestion and thrombosis (**green**) are widespread, as are the massive neutrophils (**yellow**) in both luminal epithelium and stroma sections. **B** shows that the shape of cytoplasm and nucleus is uniform in the endometrium. The positive-immunostained immune cells are shown by the arrows in **C**, **D**, **E** and **F**. Scale bars = 100 μm.

Histopathological evaluation of the H&E stained sections of the uterus of the CT dairy cows showed that thrombosis and blood vessel congestion (green arrows) were common findings, the neutrophils (yellow arrows) were extensively pronounced in the uterine endometrial epithelium and stroma, and large amounts of eosin-stained (orange arrows) secretions were found in endometrial the glandular section (Figure 5A). Noticeably, RPG supplementation reversed the changes, and the cytoplasm and nucleus were uniform in shape (Figure 5B). Immunostaining of the IGF1R and IGF2R were detected in the endometrial section. Nevertheless, higher IGF1R and IGF2R expressions were observed in the endometrium of cows fed with the RPG supplementation compared with the CT cows (Figure 5C,E). Both IGF1R and IGF2R were strongly expressed in the endometrium of cows fed with the RPG supplementation versus the CT cows (Figure 5D,F).

## 4. Discussion

Early stages of uterine involution are important because a delayed recovery in 14 d post-natal may cause the occurrence of endometritis in cows [6]. Most importantly, the rate of uterine involution can also influence the subsequent fertility of dairy cows [21,22]. In this study, RPG supplementation had no obvious effect on the uterine weight, uterine length, and uterine width of cows. The reason for this phenomenon is that the ongoing process of uterine involution at 14 d post-natal involved tissue regeneration alongside size recovery in dairy cows [6].

The nuclear localization of PCNA and Ki67 is an indication of cell proliferation [13]. To verify whether RPG promoted endometrial proliferation and healing, we investigated cell proliferation factors (PCNA and Ki67) expression by using immunofluorescence. The results confirmed that the RPG supplement significantly increased PCNA and Ki67-positive nuclei in uterus, suggesting that RPG promoted the proliferation of endometrial cells, and thereby improved uterine repair and involution following calving. 

Both IGF1 and IGF2 are constituents of the IGF system that promotes the proliferation of cells and differentiation and tissue repair [23,24]. The release of IGF1 during the early post-natal period may affect uterine health through its effect on the uterine cells or immune cells that are essential to an uterine involution [25]. The greater plasma concentration of IGF1 in the RPG cows was consistent with a previous report in which glucose infusion increased IGF1 concentration in early post-natal cows [25]. Indeed, the expression of IGF1 mRNA in the endometrium was closely related to the pregnancy of cows at 17 d [26]. The ability of IGF1 binding to receptors is modulated by the IGFBPs family with high affinity [27]. In this study, the mRNA expression of IGF1 was greater in the endometrium of cows fed with the RPG supplementation. This phenomenon suggested a beneficial uterine environment for embryo development. Additionally, the expressions of IGF1, IGF2, IGF1R, IGFBP1, IGFBP2, IGFBP4 and IGFBP5 were increased in the endometrium of cows fed with the RPG versus the CT group, and corresponding changes in the IGF1, IGF2, IGF1R, IGFBP1 and IGFBP4 protein expression occurred. These results indicated that a RPG addition regulated the bioactivities of IGFs differentially in post-natal dairy cows [6].

The IGF1 and IGF2 are also important mitogens that affect the growth and metabolism of cells [28]. The mitogenic actions of the IGF1 and IGF2 are mainly mediated through the receptor IGF1R [29]. A higher endometrial mRNA level of IGF1R in RPG fed cows indicated that more receptors were available for the IGF1 and IGF2, further heightening mitotic activities of the IGF1and IGF2 in the uterus. Although IGFBPs inhibit IGFs actions by preventing IGFs binding to IGF receptors in many circumstances [27,30], they also potentiate IGF actions. For instance, IGFBP5 stimulated the growth and remodeling of tissues independently of IGFs [6,31,32] and IGFBP2 promoted the growth of an endometrial cell directly [33]. The increased mRNA expressions of IGFBP1, 2, 5 in RPG cows than CT cows in the present study were consistent with their stimulatory effects on IGFs action [34]. Both IGFBP1 and IGFBP2 contain the amino acid Arg-Gly-Asp integrin recognition sequence, which is involved in binding to cell surfaces. The IGFBP5 also binds to the extracellular matrix [35,36]. In each case, bound IGFBP have lower affinity for IGF1 and IGF2. Therefore, the IGF1 and IGF2 were dissociated from the binding proteins in favor of the receptors [34]. Furthermore, the IGFBPs can enter the nucleus and modulate transcription by binding to nuclear receptors, ultimately enhancing IGF actions [37]. Therefore, the present study indicated that RPG supplementation might promote IGF1 and IGF2 binding to their respective receptors and enhance IGF-independent actions of IGFBPs, thereby accelerating endometrial proliferation and repair. 

The IGF1 and IGF2 can regulate the PI3K/AKT/mTOR pathway in responses to cell proliferation and tissue repair [38,39]. Studies have shown that this pathway is crucial for regulating endometrial proliferation and repair [9,40]. In the present study, the p-AKT/AKT and p-mTOR/mTOR expression levels were upregulated in cows supplemented with a RPG versus CT diet. This result suggested that RPG might promote endometrial repair by activating the AKT/mTOR pathway. 

The MMPs are another putative regulator of the IGF system in the post-natal uterus. The MMPs can regulate IGFBPs activity in which MMP3 makes IGFBP1 incapable to bind IGFs; therefore, IGF stimulated placental growth [41]. Typically, the MMPs are expressed in inflamed or injured tissues, and induced by mechanical stretch [42]. It has been reported that the MMPs are associated with tissue remodeling during uterine involution. In the rat uterus, the expression of MMP9 was significantly increased at 18 h and 36 h post-natal, but its mRNA expression was decreased significantly at 5 d after delivery [43]. The MMP3 is a matrix lysozyme and it also plays an important role in pro-MMP activation [44]. The upregulated MMP3 and MMP9 mRNA levels in the RPG group at 14 d after parturition further illustrated that RPG might be beneficial to post-natal uterine involution. It was likely that the RPG supplementation facilitated uterine involution by affecting the ability of the MMPs and altering the availability of IGFs to promote the uterine proliferation and healing in dairy cows.

## 5. Conclusions

**Figure 6:**
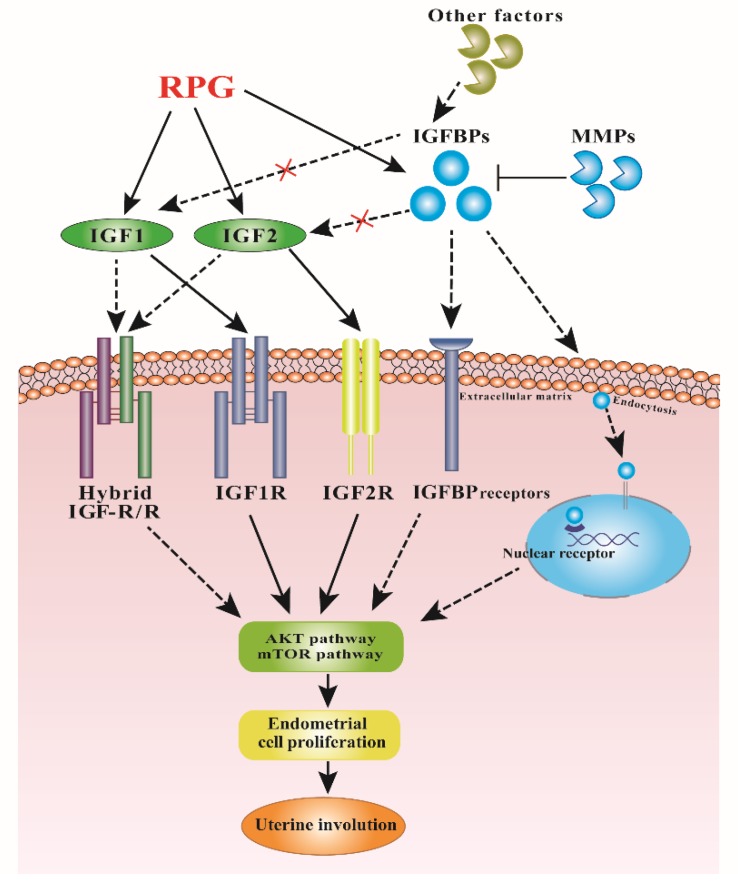
Proposed mechanism for RPG in regulating the uterine involution in post-natal dairy cows. The effect of RPG addition was mediated by both IGFs and mTOR/AKT signaling pathways. RPG addition might promote IGF1 and IGF2 binding to their respective receptors and enhance IGF-independent actions of IGFBPs, thereby activating the mTOR/AKT signaling pathway and accelerating the endometrial proliferation and repair. Each ligand displayed a specific binding affinity for the receptors: continuous arrows indicate high affinity, while dotted arrows and a red “X” indicates low affinity. The black dashed arrows are currently unknown.

In conclusion, our results revealed that RPG supplementation promoted the proliferation of endometrial cells in post-natal dairy cows by stimulating the IGF system and mTOR/AKT pathway, and might be useful in assisting the uterine involution (Figure 6). This smooth involution is of importance for an optimal transition to a new lactation and good animal welfare.

## Figures and Tables

**Table 1:** Ingredients and nutrient composition of the diets.

Item	Prepartum	Post-Natal
Ingredient (% of DM)		
Corn silage	24.7	30.2
Oat hay	55.3	12.8
Alfalfa hay	-	17.1
Ground shelled corn	5.9	7.0
Wheat bran	9.1	21.3
Soybean meal (49% CP)	4.1	8.1
Calcium carbonate	0.23	1.13
Calcium hydrophosphate	0.23	0.45
Salt	0.16	0.45
Sodium bicarbonate	-	0.68
Magnesium oxide	0.05	0.09
Potassium chloride	-	0.32
Vitamin and mineral mix ^1^	0.23	0.41
Chemical analysis, % of DM		
CP	11.6	14.6
Fat	2.0	2.1
Starch	10.3	14.8
NDF	53.6	45.2
ADF	31.3	25.5
Ash	6.8	6.0
Ca	0.50	0.98
P	0.41	0.54
NE_L_^2^, Mcal/kg	1.30	1.37

^1^ Dry cow vitamin and mineral premix (per kg): Cu 900 mg; Zn 1350 mg; Mn 1000 mg; Co 13 mg; I 27 mg; Se 23 mg; vitamin A 450 KIU; vitamin D3 110 KIU; vitamin E 5400 IU. Lactating cow vitamin and mineral premix (per kg): Cu 3040 mg; Fe 3170 mg; Zn 14,280 mg; Mn 3060 mg; Co 40 mg; I 180 mg; Se 100 mg; vitamin A 1250 KIU; vitamin D 3270 KIU; vitamin E 5000 IU. ^2^ Calculated value.

**Table 2:** The primer sequence of the target gene.

Target Genes ^1^	Primer Sequence (5’ → 3’)	Product Length, bp	Accession No. ^2^
*GAPDH*	Forward (F): ACCCAGAAGACTGTGGATGG	178	NM_001034034.2
	Reverse (R): CAACAGACACGTTGGGAGTG		
*IGF1*	F: TCCCATCTCCCTGGATTTCT	105	NM_001077828
	R: GGGTTGGAAGACTGCTGATT		
*IGF2*	F: GCTTCTACTTCAGCCGACCAT	113	NM_174087
	R:GGCACAGTAAGTCTCCAGCAG		
*IGF1R*	F: AAGGTCCTCAGCGAGTTGTTT	101	XM_606794
	R: GATCCCGTGTTCTTCTACGTTC		
*IGF2R*	F: AAGCCTCCCACTATCAACAGAA	111	NM_174352
	R: TACAACTTCCGGTGGTACACCA		
*MMP3*	F: GATGATGAACAATGGACAAAGG	134	XM_586521
	R: CGAGGGTCGTAGACTGGGTA		
*MMP9*	F: GAGGGTAAGGTGCTGCTGTTC	236	NM_174744
	R: AAGGTCACGTAGCCCACATAGT		
*IGFBP1*	F: TCAAGAAGTGGAAGGAGCCCT	127	NM_174554
	R: AATCCATTCTTGTTGCAGTTT		
*IGFBP2*	F: AATCCATTCTTGTTGCAGTTT	120	NM_174555
	R: AGGGTGGCAAACATCACCT		
*IGFBP3*	F: GAAGGCGCATGGTGGAGAT	102	NM_174556
	R: ACAGACACCCAGAACTTCTCCTC		
*IGFBP4*	F: GCCCTGTGGGGTGTACAC	342	NM_174557
	R: TGCAGCTCACTCTGGCAG		
*IGFBP5*	F: TGCGAGCTGGTCAAGGAG	257	NM_001105327
	R: TCCTCTGCCATCTCGGAG		
*IGFBP6*	F: AGAAAGAGGATTTGCCTTTGC	324	NM_001040495
	R: TCCGGTAGAAGCCCCTATG		

^1^ IGF1 = insulin-like growth factor 1; IGF1R = type 1 IGF receptor; MMP3 = matrix metallopeptidase 3; IGFBP1-6 = insulin-like growth factor binding protein 1-6; ^2^ the reference sequence number is listed for primers whose source is the National Center for Biotechnology Information (NCBI) GenBank database (http://www.ncbi.nlm.nih.gov/genbank/).

**Table 3:** The details of antibodies used for Western blot.

Antibodies ^1^	Type	Suppliers	Dilution
Primary antibodies			
p-AKT	Rabbit Polyclonal 4060	Cell Signaling Technology, Danvers, MA, USA	1/2000
AKT	Rabbit Polyclonal 9272	Cell Signaling Technology, Danvers, MA, USA	1/1000
p-mTOR	Rabbit Polyclonal 2971	Cell Signaling Technology, Danvers, MA, USA	1/1000
mTOR	Rabbit Polyclonal ab2732	Abcam, Cambridge, UK	1/1500
p-PI3K	Rabbit Polyclonal bs-5582R	Biosynthesis Biotechnology, Beijing, China	1/200
PI3K	Rabbit Polyclonal	Biorbyt, Cambridge, UK	1/300
IGF-1	Goat Polyclonal orb125044	Biorbyt, Cambridge, UK	1/500
IGF-1R	Rabbit Polyclonal ab39675	Biosynthesis Biotechnology, Beijing, China	1/500
IGF-2	Rabbit Polyclonal ab9574	Abcam, Cambridge, UK	1/2000
IGF-2R	Rabbit Polyclonal	Proteintech, USA	1/1000
IGFBP1	Rabbit Polyclonal ab231254	Abcam, Cambridge, UK	2 μg/mL
IGFBP4	Rabbit Polyclonal 483393	United States Biological, Swampscott, MA, USA	1/500
GAPDH	Mouse Monoclonal 60004-1-lg	Proteintech, USA	1/5000
Secondary antibodies			
Rabbit anti-goat IgG	SA00001-4	Proteintech, USA	1/3000
Goat anti-rabbit IgG	SA00001-2	Proteintech, USA	1/3000
Donkey anti-Mouse	SA00001-8	Proteintech, USA	1/3000

^1^ p-AKT = phosphorylated (p)-protein kinase B (AKT, Ser473); p-mTOR = phosphorylated (p)-mechanistic target of rapamycin complex 1 (mTOR, Ser2448); p-PI3K = phosphorylated (p)-phosphatidylinositol 3-kinase (PI3K, Tyr 467); IGF1 = insulin like growth factor 1; IGF1R = type 1 IGF receptor; IGFBP1 = insulin like growth factor binding protein 1; GAPDH = glyceraldehyde-3-phosphate dehydrogenase.

**Table 4:** Effects of rumen-protected glucose (RPG) on the weights of body and uterus in post-natal dairy cows.

Item	CT	RPG	*p*
Body weight (kg)	537.5 ± 65.14	486.66 ± 20.27	0.28
Uterine weight (Kg)	1.42 ± 0.36	1.72 ± 0.21	0.18
Uterine length (cm)	35 ± 5.25	35.4 ± 3.21	0.91
Uterine width (cm)	25.3 ± 3.46	29.58 ± 2.78	0.77
